# A phase i study of daily treatment with a ceramide-dominant triple lipid mixture commencing in neonates

**DOI:** 10.1186/1471-5945-12-3

**Published:** 2012-04-04

**Authors:** Adrian J Lowe, Mimi LK Tang, Shyamali C Dharmage, George Varigos, Della Forster, Lyle C Gurrin, Colin F Robertson, Michael J Abramson, Katrina J Allen, John Su

**Affiliations:** 1 Murdoch Children's Research Institute, Royal Children's Hospital, Flemington Rd, Parkville 3052, Australia; 2 The University of Melbourne, Melbourne, Australia; 3 Royal Children's Hospital, Melbourne, Australia; 4 Royal Women's Hospital, Melbourne, Australia; 5 La Trobe University, Melbourne, Australia; 6 Monash University, Melbourne, Australia

**Keywords:** Eczema prevention, Eczema, Skin barrier function, Asthma, Atopy

## Abstract

**Background:**

Defects in skin barrier function are associated with an increase risk of eczema and atopic sensitisation. Ceramide-dominant triple lipid mixture may improve and maintain the infant skin barrier function, and if shown to be safe and feasible, may therefore offer an effective approach to reduce the incidence of eczema and subsequent atopic sensitisation. We sort to assess the safety and compliance with daily application of a ceramide-dominant triple lipid formula (EpiCeram™) commencing in the neonatal period for the prevention of eczema.

**Methods:**

Ten infants (0-4 weeks of age) with a family history of allergic disease were recruited into an open-label, phase one trial of daily application of EpiCeram™ for six weeks. The primary outcomes were rate of compliance and adverse events. Data on development of eczema, and physiological properties of the skin (transepidermal water loss, hydration, and surface pH) were also measured.

**Results:**

Eighty percent (8/10) of mothers applied the study cream on 80% or more of days during the six week intervention period. Though a number of adverse events unrelated to study product were reported, there were no adverse skin reactions to the study cream.

**Conclusions:**

These preliminary results support the safety and parental compliance with daily applications of a ceramide-dominant formula for the prevention of eczema, providing the necessary ground work for a randomised clinical trial to evaluate EpiCeram™ for the prevention of eczema.

**Trial registration:**

The study was listed at the Australian/New Zealand Clinical Trial Registry (ANZCTR): reg. no. ACTRN12609000727246.

## Background

There is growing awareness of the importance of skin barrier function in the aetiology of eczema [1]. A number of genetic variants that result in null expression of filaggrin, are associated with impaired skin-barrier function, increased risk of eczema, and eczema further complicated by asthma [2,3]. The majority of childhood eczema commences within the first six months of life [4], and even healthy infants display lower skin barrier function than adults [5]. Interventions in early life that improve skin barrier could also reduce the risk of asthma and allergic rhinitis, by reduction in risk of sensitisation across a defective skin barrier [1,6] or by reducing the release of pro-inflammatory cytokines that induce airway inflammation, such as thymic stromal lymphopoietin, by eczematous skin [7].

Lipid mixtures that contain the major lipid components of human skin (ceramides, cholesterol and free fatty acids), at physiological concentrations, have been shown to greatly accelerate the restoration of barrier function (> 90% at four hours following application) in a murine model [8]. One such cream, EpiCeram™, has been demonstrated to have a similar impact on eczema severity over a 28 day treatment period as a moderate strength topical steroid cream (fluticasone propionate 0.05%) [9] in children (over six months of age) with moderate to severe eczema. Although regular ceramide-dominant treatment of neonatal skin would appear to offer the greatest likelihood of improving the skin barrier of infants, the safety and efficacy of such a cream as a preventive strategy on newborns is yet to be demonstrated.

We sought to establish the compliance of parents with daily application of EpiCeram™ to their newborn baby's skin, and to generate initial evidence that this intervention is safe for use on newborns. This information would help establish the feasibility of regular use of such an emollient to prevent eczema.

## Methods

### Trial design

This was a non-controlled (all participants treated), open label (parents of participants and study staff all knew the treatment status of all children), phase 1 study. The trial was registered with the Australian and New Zealand Clinical Trials Registry (ACTRN12609000727246, 24/8/08). Mother-baby pairs were enrolled if the parent reported that the child had a first degree relative with a history of eczema, asthma, allergic rhinitis, or food allergy. Infants were excluded if they had a parent with a known hypersensitivity to any of the ingredients of EpiCeram™, were a multiple birth, premature (< 36 weeks gestation) or were admitted to a special care nursery.

### Recruitment

Children were enrolled in the study from birth to four weeks of age between March and June 2010. A range of recruitment strategies were used: a) recruitment posters were displayed in the antenatal clinic at The Royal Women's Hospital (RWH) and the Departments of Allergy Immunology and Dermatology at the Royal Children's Hospital (RCH), Melbourne, Australia; b) RWH Pathology staff handed out a screening questionnaire when expectant mothers were attending for their routine 26 week blood tests; c) the study coordinator (Robyn Kennedy) directly approached mothers attending antenatal clinics and while they were on the maternity wards. The study coordinator assessed eligibility and enrolled participants, and all mothers provided written consent. The study was approved by both the RCH (project 20943) and the RWH (09/30) Human Research Ethics Committees.

### Intervention

The study intervention was application of EpiCeram™ to the full skin surface of the child once per day, excluding the hands and face (ingredient list and prescribing information found at http://www.epiceram-us.com/prescribing-information). EpiCeram™ contains ceramides, cholesterol and free fatty acids at a 3:1:1 ratio and 2% petrolatum, and has a low pH. The ceramides used is a pseudo (ceramide *N*-(2-hydroxyethyl)-2-pentadecanolylhexadecanamide. This formulation has been approved by the Food and Drug Administration, and has been marketed in the United States of America since October of 2008 as a treatment of eczema. To date, there is no evidence of toxicity from EpiCeram™. At the request of one of the approving ethics committees, we excluded treatment of the hands and face to minimise accidental ingestion of the cream. At the end of the baseline assessment, all mothers were given 9 × 30 gram tubes (270 g total) of EpiCeram™, and were instructed to apply approximately 6 grams (approximately a heaped teaspoon) of EpiCeram™ per application, once daily, after bathing the infant, or at a regular time each day.

### Follow-up

Parents completed a daily diary card to document application of the study cream, as well as to note any adverse events (particularly skin rash, redness or any other symptoms) that occurred. A follow-up assessment was performed at approximately six weeks (5-9 weeks) following commencement of the study cream. During this visit, the infant's skin was examined for signs of eczema, and the skin barrier function (as measured by Tewameter TM300, Courage & Khazaka, Köln), skin pH (pH-meter pH905) and skin hydration (Corneometer CM825) were assessed using standardized protocols. At this visit, participants were asked to return all tubes of study cream (including empty tubes) and these were weighed to estimate the quantity of cream used.

### Outcomes

The primary outcome was participant compliance, and frequency of adverse events. Secondary outcomes were presence of eczema, assessed using the UK working party criteria [10], parent reported doctor diagnosis of eczema, and biophysical measures of skin barrier function (TEWL), pH, and stratum corneum hydration.

### Statistical methods

The rate of compliance and the number and nature of adverse events were tabulated. No statistical comparisons were made as there was no control group.

## Results

### Participant flow

A total of 339 mothers were contacted to enroll 13 children into this study (Figure 1). Parents of three infants withdrew consent from this study following enrolment: the stated reason in each case was ongoing concern over safety of the intervention (n = 2), and/or lack of willingness by the father (n = 1) to participate. In all three cases, consent was withdrawn within seven days of enrolment and prior to application of the study cream. The remaining 10 participants attended the six week follow-up appointment.

### Participant characteristics

Anecdotally, mothers who had experienced severe allergic disease themselves, or who had an older child who had suffered from eczema, were the most interested in participating in this study. Nine of the 10 children first received the study cream within the first three weeks of life (Table 1).

**Table 1:** Characteristics of the study population, and compliance with daily application of study cream

Male infant	6/10
**Number of parents with allergic disease**	

1 parent	9/10

2 parents	1/10

**Age in weeks at:**	Median (IQR)*

commencement of treatment	2.1 (1.3-2.9)

follow-up	9.5 (7.6-10.1)

**Application of study cream - medians (range)**	

days cream applied (max = 42)	38.5 days (35-42)

proportion of eligible days applied	92% (83%-100%)

total quantity applied	158 g (150-207)

average quantity applied per day	3.8 g (3.6-4.9)

average quantity applied per day on days used	4.4 g (3.8-4.9)

*IQR interquartile range.

### Compliance

Parents of eight infants applied the study cream on 80% of the days during the intervention period (Table 1). The remaining two infants had the cream applied on 69% and 19% of days during the six week intervention period. The mother of the participant with the lowest rate of application reported that the child became distressed when undressed. There was a wide variation in the amount of study cream that was applied in total (34 - 277 grams - Table 1), and the average amount of cream that was used per application (3.6 - 7.2 grams).

### Six week follow-up

Two children showed evidence of facial eczema where the study cream was not applied. No conclusions can be drawn from the physiological data at this time due to the lack of a control group and the small sample size (Table 2).

**Table 2:** Trans-epidermal water loss (TEWL), skin pH, capacitance and sebum measured at follow up (approximately 6 weeks)

Skin parameter	Median (IQR)
TEWL (g/m^2^/hr)	
forearm	9.3 (7.2-10.5)
forehead	14.3 (8.1-25.4)

pH	
forearm	5.1 (4.8-5.4)
forehead	4.9 (4.7-5.4)

Hydration (forearm)*	45.8 (41.3-46.5)

Sebum (forehead)*	36 (22-85)

* arbitrary units of measurement

There were no cases of adverse skin reactions to the study treatment (Table 3), although a number of the infants had skin symptoms. These were either restricted to the face (where the study cream was not applied), present prior to the initial application of the cream, or noticed first on the face, and then on the child's trunk. One child developed folliculitis on his face, which is not unusual during this period of infancy in healthy infants. A number of adverse events occurred (Table 3), including one child who was hospitalised for bronchiolitis. All of these events are common conditions of infancy, and are unlikely to be related to the study treatment.

**Table 3:** Adverse events during treatment period

Skin Symptoms	Frequency	Comment
Heat rash	1	
Facial eczema	2	treatment not applied to face
Folliculitis	1	started on face - non treated area - and spread to trunk
Dry skin (torso, ankles, wrists)	1	present prior to first application

**Other symptoms**		

Bronchiolitis	1	resulted in hospitalisation
Cough/fever	1	
Conjunctivitis	2	
Reflux	1	
Cold/flu	3	

## Discussion

In this phase-1 trial, most parents applied the ceramide-dominant triple lipid mixture to the skin of their child on most days during the study period, and there were no major adverse reactions attributable to the intervention. Parents were also willing to start applying the cream within the first 2-3 weeks of life, when the skin barrier is most compromised even in health infants [5]. Due to the lack of a control group, no conclusions can be made concerning the effect of the intervention on skin barrier, although the obtained TEWL values appear to be substantially lower than those reported previously in this age group (mean = 9.3 g/m^2^/hr, range = 7.2-10.5 for forearm for the current study compared with mean = 26 g/m^2^/hr for infants aged 3-6 months in a prior publication [5]). Adverse events that occurred were common conditions in this age group and did not appear to be related to the study treatment.

There are a number of other phase 1 and 2 trials currently being conducted to evaluate various topical interventions for improving the skin barrier function of infants at increased of developing eczema. One similar study is evaluating the effects of a standard emollients (Cetaphil, an oil-in-water cream) [11]. We have chosen an emollient that contains ceramides, cholesterol and free fatty acids at a 3:1:1 ratio (which also contains 2% petrolatum) and has been formulated to correct the underlying lipid defects in eczematous stratum corneum [12]. There is limited data available at this time [9,13] comparing EpiCeram with cheaper emollients. A recent study has found that the efficacy of EpiCeram was no-greater than an over the counter petrolatum-based moisturiser (Aquaphor) for the management of mild-to-moderate atopic dermatitis, and that the cost effectiveness was far greater for the over the counter formulation [13]. It should be noted that the previous study assessed the effects of these treatments for the management of symptoms for existing eczema, rather using it as a preventive strategy. Secondly, the observed results were obtained by applying the treatments three times per day [13]. It would appear unlikely that parents would be able, or willing, to undertake such an intensive skin care regime for the prevention of eczema.

A potential role for EpiCeram in eczema prevention is supported by findings that: ceramide profile correlates with eczema [14], and applied ceramides rapidly incorporate into the nucleated layers of the epidermis [15]. Furthermore, EpiCeram is formulated at an acidic pH (5.0), as lamellar body production and secretion of ceramides is impaired at the high pH of eczematous skin [16]. Also, EpiCeram contains 2% petrolatum, which provides similar benefits of pure petrolatum, but without the greasiness [15], which is an important consideration for an eczema prevention strategy.

No adverse reactions to the study cream occurred in this small sample of infants. Although concerns have been raised about the toxic potential of excess ceramides, the pseudo-ceramide used in EpiCeram™ do not share these properties [17]. There have been no reported events of toxicity caused by EpiCeram™ in the three years of marketing this product as a treatment for eczema. One of our institutional ethics committees questioned the application of this intervention to the face and hands of infants, due to the risk of ingestion of the cream. However, given that eczema generally commences on the scalp, face and neck [18], and that two of the infants developed symptoms of facial eczema, it will be important to apply the cream to the full body surface, including the face and hands, in future studies. Although the small amount of cream that can be ingested is unlikely to cause adverse events, given the novelty of this intervention and the vulnerability of the participant population, we will continue to monitor the safety of this intervention, including systemic and gastrointestinal reactions.

Importantly, this study provided useful information on recruitment strategy. Recruitment into this trial proved surprisingly difficult, as demonstrated by the high number of mothers approached to achieve 10 children completing this study. We also found a reasonably high rate (3/13 parents) who initially consented for their child to be enrolled in the study, but soon after decided to withdraw from the study, which indicates that "over recruitment" will need to be used in similar future studies. We trailed a range of potential recruitment strategies, finding that a post-natal approach was most efficient, due to the higher rate of recruitment, and minimising the delay from the expression of interest to the initiation of treatment. Anecdotally, we also observed that parents of children with the strongest family history of allergic disease, particularly those who had an older child with moderate to severe eczema, were most interested in being involved in this type of study.

This study has two important limitations. Firstly, although we did not observe any adverse skin effects of the study treatment, the relatively sample size means that we cannot exclude the possibility that this treatment may cause rarer adverse effects. Secondly, the lack of a control group precludes us from assessing if this treatment may help improve skin barrier function and reduce the risk of eczema. We are currently developing further trials to address these limitations.

## Conclusions

The use of ceramide-dominant triple-lipid formulation on the skin of starting during the neonatal period is a novel approach to eczema and atopic disease prevention. The treatment used in this study, EpiCeram, has been approved by the Food and Drug authority for the treatment of eczema. This pilot study supports the safety and parental compliance with daily applications of this formulation for the skin of neonates, for the purpose of possible eczema prevention. However, recruitment remains problematic. We are currently initiating a phase-2/3 trial of this intervention, and we hope that this intervention will improve infant skin barrier function sufficiently to reduce the incidence of eczema and atopic disease. Future studies will need to also assess the cost-effectiveness of such strategies, including head-to-head evaluations with cheaper, over-the-counter, formulations.

## Competing interests

Ceragenix providing EpiCeram™ for this study, free of charge. All authors declare that they have no other competing interests.

## Authors' contributions

AJL, JS, SD & MLKT designed the study. AL drafted the protocol, and all authors reviewed and provided critical comment. AJL supervised study-staff. All read and approved the final manuscript.

## Declaration of all sources of funding

This study was supported by a Fred Bauer Project Grant from the Australasian College of Dermatology. Ceragenix provided the study intervention (EpiCeram™) without cost to the study. AL, SD and LG are currently supported by the Australian National Health & Medical Research Council. KA is supported by a Sylvia Viertal Fellowship.

## Pre-publication history

The pre-publication history for this paper can be accessed here:

http://www.biomedcentral.com/1471-5945/12/3/prepub
